# Prediction of daily new COVID-19 cases ‐ Difficulties and possible solutions

**DOI:** 10.1371/journal.pone.0307092

**Published:** 2024-08-23

**Authors:** Xiaoping Liu, A. Courtney DeVries

**Affiliations:** Department of Medicine, Department of Neuroscience, Rockefeller Neuroscience Institute, West Virginia University Health Science Center, Morgantown, West Virginia, United States of America; University of Rajshahi, BANGLADESH

## Abstract

Epidemiological compartmental models, such as *SEIR* (Susceptible, Exposed, Infectious, and Recovered) models, have been generally used in analyzing epidemiological data and forecasting the trajectory of transmission of infectious diseases such as COVID-19. Experience shows that accurately forecasting the trajectory of COVID-19 transmission curve is a big challenge for researchers in the field of epidemiological modeling because multiple unquantified factors can affect the trajectory of COVID-19 transmission. In the past years, we used a new compartmental model, *l-i SEIR* model, to analyze the COVID-19 transmission trend in the United States. Unlike the conventional *SEIR* model and the delayed *SEIR* model that use or partially use the approximation of temporal homogeneity, the *l-i SEIR* model takes into account chronological order of infected individuals in both latent (*l*) period and infectious (*i*) period, and thus improves the accuracy in forecasting the trajectory of transmission of infectious diseases, especially during periods of rapid rise or fall in the number of infections. This paper describes (1) how to use the new *SEIR* model (a mechanistic model) combined with fitting methods to simulate or predict trajectory of COVID-19 transmission, (2) how social interventions and new variants of COVID-19 significantly change COVID-19 transmission trends by changing transmission rate coefficient *β*_*n*_, the fraction of susceptible people (*S*_*n*_/*N*), and the reinfection rate, (3) why accurately forecasting COVID-19 transmission trends is difficult, (4) what are the strategies that we have used to improve the forecast outcome and (5) what are some successful examples that we have obtained.

## Introduction

The World Health Organization (WHO) has declared an end to the COVID-19 global health emergency on May 5, 2023 [1]. The COVID-19 pandemic has greatly damaged the health of the people in the world. Over the past four and a half years, there were more than 7 million people who died from COVID-19 globally [2]. COVID-19 is caused by SARS-CoV-2 virus that is prone to mutations and the generation of genetic variants [3]. Since its first outbreak in 2019, SARS-CoV-2 has continually evolved, resulting in the emergence of several prominent variants, including Alpha, Beta, Delta, and Omicron that have gained more efficient transmission, severity, and immune evasion properties. The latest SARS-CoV-2 variant, Omicron, had the strongest breakthrough infectivity and re-infectivity compared to the previous SARS-CoV-2 variants [4–6].

Throughout the pandemic, researchers used mathematical models to analyze COVID-19 data for better understanding transmission patterns, monitoring disease severity, anticipating future epidemic outcomes [7] and justifying the adoption of intervention measures [8]. Among these mathematical models, compartmental models describing the disease as a sequence of different stages encountered upon infection to recovery, such as *SEIR* (*S*usceptible-*E*xposed-*I*nfectious-*R*ecovered) model, have been generally adopted to forecast or simulate future transmission trajectories [9, 10]. These compartmental models provide a parsimonious (i.e., using few parameters) approach to understanding important behaviors of epidemic pathways. Experience has shown that such models generate robust results that strengthen their usefulness [11]; however, it has been recognized that forecasting COVID-19 transmission trajectories remains a big challenge to the mathematical modelers [7, 9, 11, 12]. The conventional compartmental models [13, 14] assume that infected individuals in each related compartment have no temporal heterogeneity, so all infected individuals in a compartment have the same probability to transfer to their next compartment. However, the realty is different: individuals are usually infected on differential days with a chronological order, so on average, individuals infected earlier in one compartment will be transferred to their next compartment at an earlier time. To describe the effects of disease latency, time delays have been included in these compartmental models, such as *SIR* and *SEIR* models [15–17]. However, these time delayed models did not completely solve the problem related to temporal heterogeneity of infected individuals, because the rate of the infectious individuals (*dI*(*t*)/*dt*) exiting from compartment *I* is still proportional to *I*(*t*) (see Eqn (1.1) in Hattaf’s paper, Eqn (15) in Huang’s paper, and Eqn (2.4) in Cooke’s paper). In our recent paper about *l-i SEIR* model, we have demonstrated that the terms proportional to *E*(*t*) or *I*(*t*) are major contributors to calculation errors due to the assumption of temporal homogeneity underlying these two terms [18]. The *l-i SEIR* model takes into account of temporal heterogeneity or the chronological order of infected individuals in both period *l* (latent period or compartment *E*) and period *i* (infectious period or compartment *I*) based on the first-in, first-out rule. It was demonstrated that, when calculating the transfer rate of infected individuals from one compartment of the *SEIR* model to the next compartment, the temporal homogeneity approximation in the conventional *SEIR* model leads to calculation errors that increase linearly with the rate of change in the number of infectious individuals. Despite the improvement in calculation accuracy of the *SEIR* model after taking into account of the chronological order of infected individuals in the model, multiple other factors (such as interventions on social distancing, face masks, vaccination, and the emergence of new, more contagious COVID-19 variants) may still affect the accuracy of prediction from compartmental models [7, 9, 11]. Understanding how these factors affect forecast results is important to improving forecast accuracy.

In a review article, Holmdahl and Buckee [9] described forecasting models, mechanistic models and hybrid approaches in modeling studies of COVID-19 transmission. Forecasting models typically fit a line or curve to data and extrapolate from there. In contrast, mechanistic models, like *SEIR* model, mimic the way COVID-19 spreads and can be used to simulate future transmission scenarios under various assumptions. There are hybrid approaches, such as the one we will cover in this paper, fitting a curve calculated from the *l-i SEIR* model to reported COVID-19 data and extrapolating the calculated curve to forecast the trajectory of COVID-19 spread in the future. We will describe the difficulties that we have encountered in predicting transmission trajectories of COVID-19 from the *l-i SEIR* model and some strategies that we have used to overcome these difficulties. Because Omicron became the dominant SARS-CoV-2 strain in the U.S. since late December, 2021 [19], our data analysis mainly focuses on the data of COVID-19 transmission caused by omicron in the United States, covering the whole period from the early outbreak of COVID-19 Omicron in the US to May 5, 2023 when data of daily COVID-19 cases in the US were not updated anymore on websites. As the final result in this study, we demonstrated our accurate prediction of the trajectory of daily COVID-19 cases in the US, which was documented in the public website (Twitter, now known as X), over a nearly 3-month period from February 10, 2023, to May 5, 2023. By searching PubMed, we have not found a similar study like this. We hope that this paper will be helpful for researchers to share their experiences in predicting the spread of infectious diseases.

## Methods

**Fig 1:**
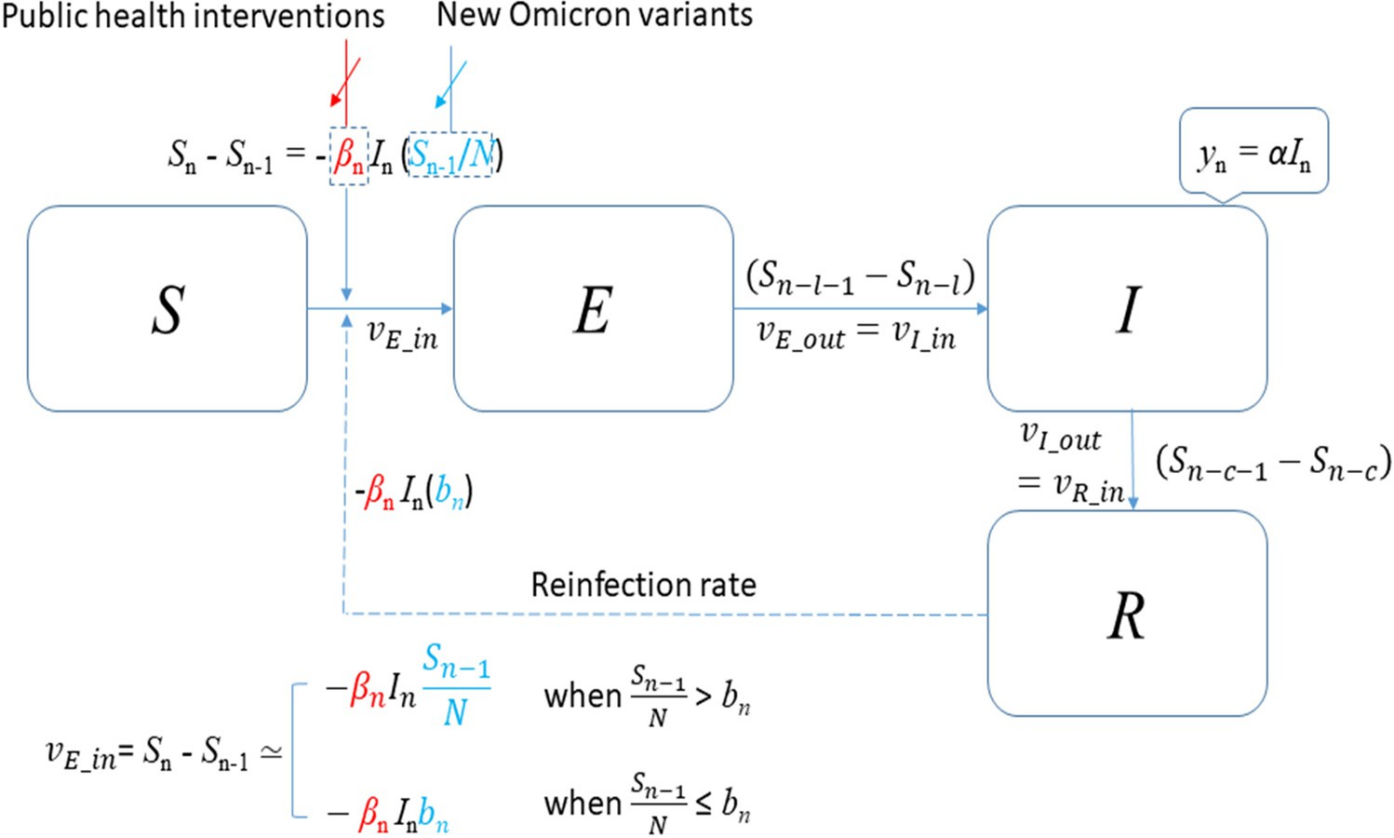
Transmission dynamics of COVID-19 Omicron described by the *l-i SEIR* model.

The transmission dynamics of Omicron-caused COVID-19 described by the *l-i SEIR* model in this study is demonstrated in Fig 1. In this section, we present *l-i SEIR* model equations considering and ignoring Omicron-to-Omicron reinfection and describe the procedures for determining parameters and coefficients in these equations.

### The *l-i SEIR* model with or without Omicron-to-Omicron reinfections

Because the latest SARS-CoV-2 variant, Omicron, has strong breakthrough infectivity and re-infectivity to the previous COVID-19 variants [4–6], we hypothesized that during the early stages of an Omicron-caused COVID-19 outbreak, most people who were not previously infected with Omicron were susceptible to Omicron infection. Furthermore, we hypothesized that the Omicron reinfection after Omicron-caused infections (or Omicron-to-Omicron reinfection) was ignorable in the first big wave of the Omicron-caused COVID-19 outbreak. This hypothesis was consistent with the later report that the mean time for Omicron-to-Omicron reinfections after the initial infection needs about 22 weeks (or 5 months) [20], which is significantly longer than the time frame (around 2 months from late December 2021 to late February 2022) of the first big wave (outbreak) of Omicron-caused COVID-19 transmission in the USA. If Omicron-to-Omicron reinfection can be ignorable, the *l-i SEIR* (*S*usceptible-*E*xposed-*I*nfectious-*R*ecovered) epidemic model for the Omicron-caused COVID-19 transmission can be described by the following recursive equations:

$$S_{n}-S_{n-1}=-\beta_{n}I_{n}\frac{S_{n-1}}{N}$$ (1a)

$$E_{n}-E_{n-1}=\left(S_{n-1}-S_{n}\right)-\left(S_{n-l-1}-S_{n-l}\right)$$ (1b)

$$I_{n}-I_{n-1}=\left(S_{n-l-1}-S_{n-l}\right)-\left(S_{n-c-1}-S_{n-c}\right)$$ (1c)

$$R_{n}-R_{n-1}=\left(S_{n-c-1}-S_{n-c}\right)$$ (1d)

To connect the calculated model variable with the daily new COVID-19 cases, we assume:

$$y_{n}=\alpha I_{n}\approx {\bar{y}}_{n}$$ (1e)

Definitions of all variables, parameters, and coefficients in Equation 1 are listed in Table 1. In Eqn (1a), *N* is the number of susceptible people right before the infectious disease spreads out. If all people in the population are susceptible to the infectious agents before the infectious disease spreads out, *N* equals to the number of population *P*. However, if a portion of people has immunity to the infectious disease before the infectious disease spreads out, *N* is smaller than *P*. For the initial condition of Eqns (1a)–(1d), we assume: (a) *S*_*n*_ *= N* and *E*_*n*_ = *I*_*n*_ = *R*_*n*_ = 0 as *n* < 0; and (b) *S*_0_ *= N-*1, *E*_0_ = 1, *I*_0_ = *R*_0_ = 0. Eqns (1a)–(1d) were derived based on the following assumption: In the outbreak period of Omicron-caused COVID-19, change in *S*_n_ is proportional to *I*_*n*_ and proportional to *S*_*n*_/*N*, and Omicron-to-Omicron reinfection can be ignorable. Under this assumption, both *S*_*n*_/*N* and the number of exposed individuals entering compartment *E* per day $\left(\beta_{n}I_{n}\frac{S_{n-1}}{N}\right)$ will gradually approach 0 in the later stages of COVID-19 spread if no effective public health interventions (such as wearing masks, social distancing, and quarantine) were implanted during the pandemic.

**Table 1:** Variables, parameters, and coefficients in Equation 1.

Variables, parameters & coefficients	Definition
n	Number of days passed since the day (n = 0) on which the first person was exposed
*S*_n_	Number of remaining susceptible individuals who are able to contract the disease on day n
*E*_n_	Number of exposed individuals who are in the latent period before becoming infectious on day n
*I*_n_	Number of infectious individuals who are in the infectious period and are capable of transmitting the disease on day n
*R*_n_	Number of people who have recovered and developed immunity on day n
*β*_*n*_	The transmission rate coefficient on day n
*l*	The average time length of latent period
*i*	The average time length of infectious period
*c*	The sum of the average time length of latent period (*l*) and infectious period (*i*)
*N*	Total number of susceptible people right before the infectious disease spreads out
*α*	Transient incidence of the infectious people, which is a fraction between 0 and 1.
*y*_n_	Calculated number of the daily confirmed COVID-19 cases
$\bar{y}$ _n_	Reported number of the daily confirmed COVID-19 cases
*P*	Population
*b*_*n*_	Final ratio of the remaining number of susceptible people to the total number of susceptible people (*N*)

The mathematical model expressed by Eqns (1a)–(1e) describes the transmission process of Omicron-caused COVID-19 without considering Omicron-to-Omicron reinfections. However, in reality, the Omicron-to-Omicron reinfection rate is not zero but a number that cannot be ignored in the later period of Omicron-caused COVID-19 spread although the Omicron-to-Omicron reinfection rate is small. Our data analysis on Omicron-caused COVID-19 spread shows that taking account of Omicron-to-Omicron reinfection rate, the number of exposed individuals who enter latent period (or compartment *E*) per day can be expressed as *β*_*n*_*l*_*n*_*b*_n_, where *b*_*n*_ is a non-zero constant. To simplify the calculation program of *E*_n_ and *I*_n_ in Eqns (1b) and (1c), we still use (*S*_n_ -*S*_n-1_) to represent the number of exposed individuals entering compartment *E* per day when the Omicron-to-Omicron reinfection rate cannot be ignored, but Eqn (1a) is replaced by the following Equation:

$$S_{n}-S_{n-1}=-\beta_{n}I_{n}\left[\frac{S_{n-1}(\geq 0)}{N}+b_{n}\frac{N-S_{n-1}(\geq 0)}{N}\right]$$ (1a)’

In Eqn (1a)’, *S*_*n*_ (≥0) = *S*_*n*_ if *S*_*n*_>0, and *S*_*n*_ (≥0) = 0 if *S*_*n*_≤0. The second term in the square brackets consists of a reinfection rate coefficient *b*_*n*_ (0≤*b*_*n*_≤1) and a weight factor (*N*- *S*_*n-*1_(≥0))/*N*. When *S*_*n*_ is close to *N*, the weight factor is close to 0 and the first term in the square brackets plays the main role. However, when *S*_*n*_ is much smaller than *N*, the weight factor is close to 1 and the second term in the square brackets plays the main role. If *b*_*n*_ = 0, it means no Omicron-to-Omicron reinfection. In this situation, *S*_*n*_ can vary from *N* (no one is infected) to 0 (all susceptible people are infected). If Omicron-to-Omicron reinfection rate is non-negligible, then *b*_*n*_ is greater than 0. In this situation, *S*_*n*_ can vary from *N* (no one is infected) to a negative number. The negative number means that not only are all susceptible people infected, but also some of them are re-infected.

### Estimation of parameters and coefficients in the model equations

After mid-December 2020, COVID-19 vaccines were given to people in the US. Since then, COVID-19 vaccines gradually became an important factor to affect the trajectory of COVID-19 transmission. In 2021, the COVID-19 alpha variant emerged in the USA and caused a transmission peak in mid-April; and then the delta variant emerged in the USA and caused a transmission peak in early September [21]. In this situation, multiple factors including vaccination (affect *S*_0_), breakthrough infection [5] (affect *S*_n_), reinfection [4] (affect rate equations), and intervention measures (affect transmission rate coefficient *β*_*n*_) were able to affect the trajectory of *y*_n_, making simulations or predictions of *y*_n_ trajectory more complicated because the coexistence of these factors made it difficult to identify who were susceptible and who were immune in the US. This complicated situation changed when the Omicron variant of COVID-19 virus began to spread. The Omicron variant had the strongest breakthrough infectivity and re-infectivity compared to the other previous COVID-19 variants [4–6]. Vaccine effectiveness to omicron, comparing to Delta variants, dropped from 0.52 to 0.38 for those who had had their second dose 180 days earlier or more [6]. Considering that many people in the US have only received one dose or even have not received vaccines, the actual number of people with immunity to omicron variant may be less than 38% (0.38) of the US population (*P* = 330,000,000). Our simulations show that the transmission of omicron variant in the US can be treated as the transmission of a new infectious disease from the beginning by assuming that only a fraction ~0.25 of the US population has immunity to the Omicron original variant in the early period of Omicron-caused COVID-19 outbreak in the US. This indicates that *N* is ~75% of the population *P* (*N≈*0.75*P* = 250000000). Here *N* = 0.75*P* is an estimated average number of susceptible individuals at the start of the COVID-19 outbreak in the United States due to the original Omicron variant, while the remaining 0.25*P* (or *P-N*) is the estimated average number of people in the United State who are immune to the original Omicron variant. Actual situation is more complicated: A portion of the people classified as susceptible may already have some immunity, albeit lower, to the original Omicron variant. Likewise, individuals classified as immune to the original variant of Omicron may not be 100% immune to the original variant of Omicron.

When we used the *l-i SEIR* model to simulate and predict the daily new COVID-19 cases, the first important thing being recognized was that the transmission rate coefficient *β*_*n*_ of COVID-19 varies with time [22, 23]. The coefficient *β*_n_ represents the efficiency of the interaction between *I*_*n*_ and *S*_*n*_/*N*. During outbreak of COVID-19, including Omicron-caused COVID-19, some public health interventions (such as maintaining a relatively large social distance between people, wearing face masks, and staying at home) were generally used to reduce COVID-19 transmission rate. These interventions reduced the transmission rate coefficient *β*_*n*_ by lowering the efficiency of the interaction between *I*_*n*_ and *S*_*n*_/*N*. Thus, *β*_*n*_ varies with time especially during COVID-19 outbreak. In studies of COVID-19 transmission, the time-dependent transmission rate coefficient has been also recognized by other researchers recently [24–32]. To simulate the transmission process of Omicron variants, we first estimated the initial value of transmission rate coefficient *β*_n_ from the reported number of daily new COVID-19 cases before Omicron started to spread out in the US by using the method described previously [18, 33]. The related computation program can be found in the worksheet “time-dependent rate” of the Excel file [34]. This estimated initial value of *β*_*n*_ (*β*_*n*_ = 0.7) combining with other estimated or determined parameters and coefficients (such as *l*, *i*, *α* and *N*) were used for calculating/simulating Omicron-caused daily COVID-19 cases.

We previously demonstrated how to obtain the values of *l*, *i* and *α* of *l-i AIR* model from the daily new COVID-19 cases reported in early 2020 when the COVID-19 outbreak began in the US [23, 33]. The *l-i SEIR* model is another form of the *l-i AIR* model [18], and the two models can be converted to each other with the same set of parameters *l*, *i* and *α* [34]. Thus we can use the same method for *l-i AIR* model described previously [33, 35] to determine the parameters *l*, *i* and *α* for *l-i SEIR* model. Briefly, we first plotted logarithm of ${\bar{y}}_{n}$ (7-day average of daily reported new COVID-19 cases), log(${\bar{y}}_{n}$), in the early period of COVID-19 outbreak (See Table 1 in [33]) vs date (or n), which formed a straight line with a slope *k*_0_ = 0.1368. Then, we calculated *y*_n_ from Eqn (1) of the *l-i AIR* model [33] or above Eqn (1) of *l-i SEIR* model for a given pair of parameters *l* and *i* assuming that *β*_n_ = 1 and *α* = 1 under the condition that the number of the total infections is much less than *N* (or *S*_n_/*N*≈1). Plot of logarithm of *y*_n_, log(*y*_n_), vs n would also form a straight line for the given pair of *l* and *i*. In this way, we could obtain the slope *k*(*l*,*i*) of the straight line for any given pair of *l* and *i* (see Table 2 in [33] and the related calculation program in [35]). When a pair of *l* and *i* makes the slope *k*(*l*,*i*) to be closest to *k*_0_, this pair of *l* and *i* was chosen to be used in the *l-i AIR* or *l-i SEIR* model for simulating an epidemic curve of COVID-19. It can be seen that when *l* = 4 and *i* = 10, the slope of the plot of log(*y*_n_) vs date is 0.1372 (or *k*(4,10) = 0.1372), which is closest to the slope *k*_0_ = 0.1368 of the plot of log(${\bar{y}}_{n}$) vs date. However, the intercepts of the two straight lines (log(*y*_n_) vs date and log(${\bar{y}}_{n}$) vs date) may have large difference. By selecting a suitable date for the first non-travel-related COVID-19 case in the US and regulating the value of *α*, we could change the intercept of the plot of log(*y*_n_) vs n. In this way, we could find a value of *α*, which minimizes the difference between the two straight lines (log(*y*_n_) vs n and log(${\bar{y}}_{n}$) vs n), by the least squares method. It was found that when *α* = 0.01453 and the first non-travel–related U.S. case (the first contagious person in the USA) was assumed to begin on February 6, 2020, which is 3 days earlier than the date that we estimated for New York City [23]. These estimated first-case-starting dates are within the time range suggested [36, 37]. The procedure for determining *l*, *i* and *α* of *l-i AIR* model was previously described in detailed [33] and the related calculation programs in Excel can be found in Mendeley Data repository [35].

**Table 2:** The determined time-dependent *β*_n_ *N* = 250000000.

Dates	*β*_n_
Dec. 25, 2021	0.4
Dec. 27, 2021	0.3
Dec. 29, 2021	0.25
Jan. 2, 2022	0.22
Jan. 4, 2022	0.2
Jan. 9, 2022	0.17

The coefficient α in *l-i SEIR* model is defined as the transient incidence rate of the infectious people, and α is related to the procedure used for confirming a COVID-19 case when we study COVID-19 transmission. In general, α may vary with time. We observed significant changes in α when analyzing the spread of COVID-19 in Wuhan in early 2020. This significant change in α is mainly caused by the use of some special interventions, such as a substantial increase in the number of viral tests and the use of 16 Fangcang shelter hospitals to admit a large number of COVID-19 infections [23]. However, our data analysis on New York city, New York State and the United States show that α is near a constant, which is 0.01176 in New York City and New York State [23], and 0.01453 in the USA [18]. Assuming that α is a constant in the USA, we calculated the cumulative number of COVID-19 infections, including asymptomatic infections, in the USA from late February, 2020 to September 30, 2020. The calculated number on September 30, 2020 is very close to the real number of infections (including asymptomatic COVID-19 infections) in the USA reported on September 30, 2020 [18], suggesting that the parameters used in the model and assuming α to be a constant in the USA are reasonable.

## Results and discussion

The following data analysis mainly focuses on the data of COVID-19 transmission caused by omicron in the United States from the early outbreak of COVID-19 Omicron in the US (late 2021) to May 5, 2023, when data of daily COVID-19 cases in the US were not updated anymore on websites.

### Predicting the peak height and the peak date of reported daily COVID-19 cases (${\bar{y}}_{n}$)

COVID-19 is highly contagious. Public health interventions are generally used to reduce the transmission rate coefficient *β*_n_ during COVID-19 outbreak. As a result, *β*_n_ may gradually decrease before the reported number of daily new COVID-19 cases ($\bar{y}$_*n*_) reaches its peak. Because the quantitative relationship between these interventions and values of *β*_n_ is unknown and transmission rates of COVID-19 in the early outbreak period are highly sensitive to *β*_n_, it is difficult to accurately predict the peak date (the date when $\bar{y}$_*n*_ reaches its peak) and peak height of $\bar{y}$_*n*_ with inaccurate values of *β*_n_. In the following, we will do a series of simulations to examine how an inaccurate *β*_n_ affect the magnitude of errors in predicting the peak height and the peak date of $\bar{y}$_*n*_. Furthermore, we will explore how to predict $\bar{y}$_*n*_ peak based on these simulations.

**Fig 2:**
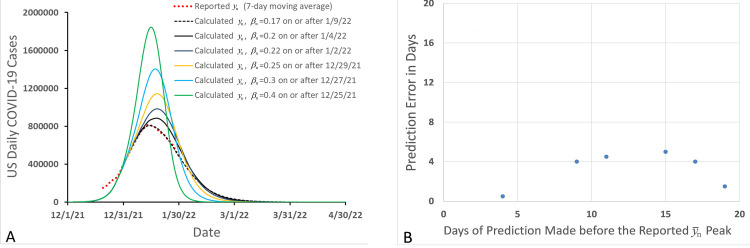
Prediction errors in forecasting peak height (A) and peak day (B) of Omicron-caused daily new COVID-19 cases in the United States.

In the simulations, we assumed that we were in the early outbreak period of Omicron-caused COVID-19, where ${\bar{y}}_{n}$ was rising rapidly before reaching a peak and we hoped to use *l-i SEIR* model and the latest available ${\bar{y}}_{n}$ data at that time to predict height and date of ${\bar{y}}_{n}$ peak. Furthermore, we had: *l* = 4, *i* = 10 [18, 23, 33], *α* = 0.01453 [18, 23], *β*_0_ = 0.7 and *N* = 250,000,000. By regulating *β*_n_, we could fit the calculated *y*_*n*_ to the latest reported ${\bar{y}}_{n}$ data. In this way, we could determine the value of *β*_n_ on the latest date. For example, assuming that today is December 26, 2021 and that we have known all ${\bar{y}}_{n}$ as of December 25, 2021, we can determine all values of *β*_n_ on or before December 25, 2021 by fitting the calculated *y*_*n*_ from the Eqns (1a)–(1e) to the reported ${\bar{y}}_{n}$ as described previously [18, 23, 33]. The value of *β*_n_ determined on December 25, 2021, is listed in Table 2 and other determined values of *β*_n_ before December 25, 2021 are not listed. With these determined values of *β*_n_, we couldn’t accurately predict when ${\bar{y}}_{n}$ would reach its peak and what would be the height of ${\bar{y}}_{n}$ peak because we didn’t know the accurate values of *β*_n_ after December 26, 2021. To make a prediction about the trajectory of ${\bar{y}}_{n}$ in the near future from December 26, 2021, we assumed that *β*_n_ after December 26, 2021, was a constant, the same as the value determined on December 25, 2021. Thus, we could simulate a trajectory of *y*_n_ from Eqns (1a)–(1e) to see the peak date and peak height of COVID-19 transmission wave as shown in Fig 2A (the green solid line, peaking on January 15, 2022, with a peak height of 1.84 million cases/day). Repeating this process, we could obtain values of *β*_n_ on the later dates (from December 26, 2021 to January 9, 2022) as shown in Table 2 and simulated corresponding trajectories of *y*_n_ as shown in Fig 2A. In this way, we forecasted the height and date of $\bar{y}$_*n*_ peak on different days before the real $\bar{y}$_*n*_ peak appeared. The *y*_n_ peak predicted on December 25, 2021 is 1.84 million, which has the largest error in comparison to the value of the reported peak $\bar{y}$_n_ (0.81 million) on January 13 and 14 in the year 2022. As the prediction day (the day on which the prediction of *y*_n_ peak is made) approaches to January 13, 2022, the predicted height of peak *y*_n_ approaches to the actual reported height of peak $\bar{y}$_n_ (Fig 2A). Thus, although it is difficult to accurately predict the height of $\bar{y}$_n_ peak at early stage of COVID-19 outbreak because of the continuously varied *β*_n_, the prediction accuracy will be significantly improved if the latest-determined *β*_n_ is used when the prediction day approaches the date of the reported $\bar{y}$_n_ peak. Usually, the height of *y*_*n*_ peak predicted on a day, during the rising phase of the actual $\bar{y}$_n_ peak and much earlier than the date of the actual $\bar{y}$_n_ peak, may be significantly greater than the actual height of $\bar{y}$_n_ peak; therefore, the height of $\bar{y}$_n_ peak predicted in this way can be considered as an estimated upper limit of the height of the $\bar{y}$_n_ peak. In contrast, simulations (Fig 2B) show that the largest error in predicting the date of $\bar{y}$_n_ peak occurs on the day that is about 15 days before the date of $\bar{y}$_n_ peak, and that an earlier prediction date may not cause a larger error in predicting the date of the $\bar{y}$_n_ peak. Thus, the date of $\bar{y}$_n_ peak may be predicted within a limited (less than 5 days in Fig 2B) error with this *l-i SEIR* model during the early rising phase of the COVID-19 outbreak, two to three weeks before the date of $\bar{y}$_n_ peak.

In the prediction of $\bar{y}$_n_ peak described above, we assumed that the time-dependent transmission rate coefficient *β*_n_ became a constant on and after the day that the prediction of $\bar{y}$_n_ peak was made. In this way, we can predict an upper limit of the height of the $\bar{y}$_n_ peak and predict the date of the $\bar{y}$_n_ peak within a limited error. One could also propose a linear or non-linear technique to extrapolate future values of the time-dependent *β*_n_, which may improve the accuracy in predicting the height of $\bar{y}$_n_ peak (for a short period depending on the prediction accuracy of *β*_n_) [38].

### Predicting the trajectory of ${\bar{y}}_{n}$ after ${\bar{y}}_{n}$ peak

**Fig 3:**
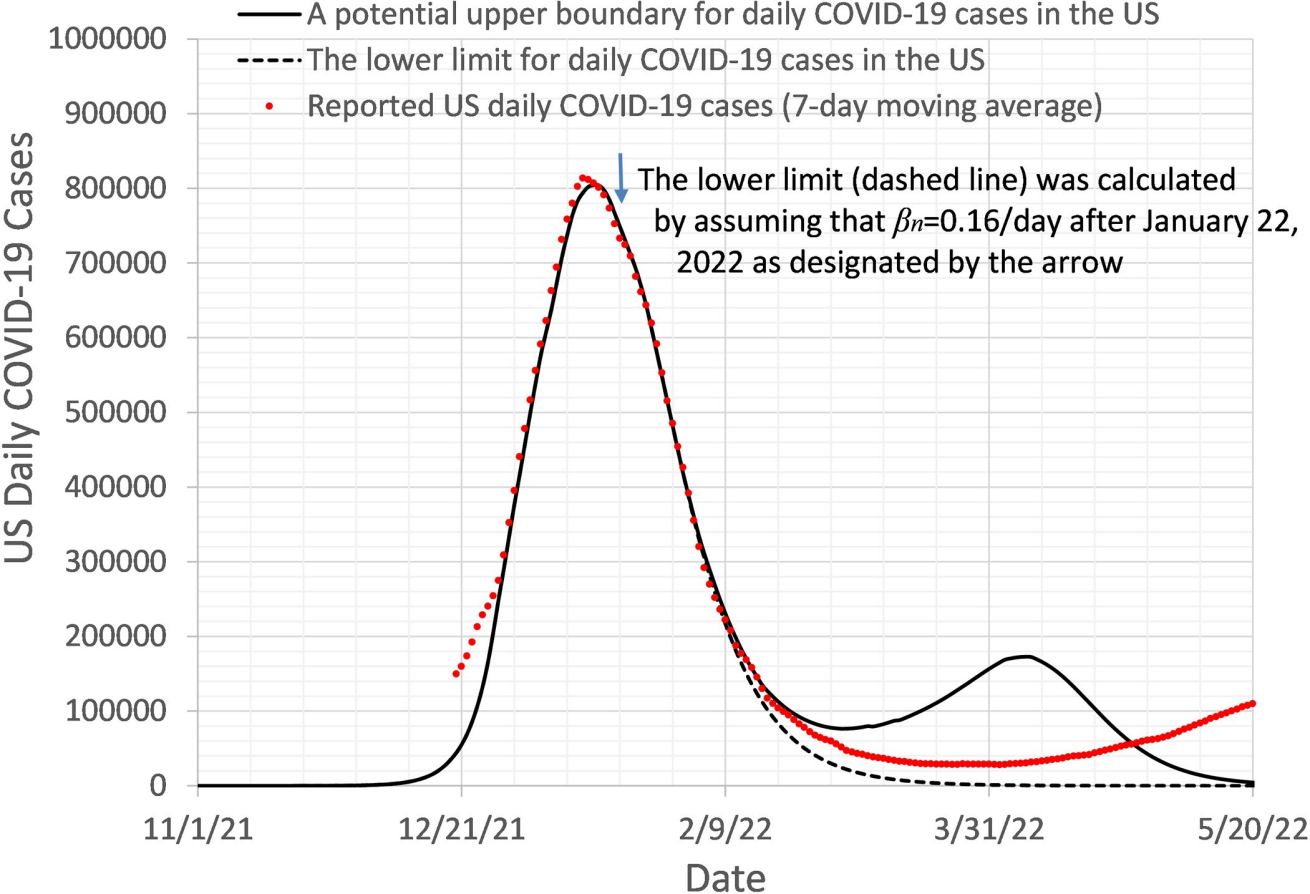
The simulated lower limit (dashed line) and upper limit (solid line) of $\bar{y}$_n_. The reported $\bar{y}$_n_ (red dotted line) is on or between the simulated lower and upper limits of $\bar{y}$_n_.

After the reported number of daily new COVID-19 cases, $\bar{y}$_n_, passes its peak, *β*_n_ may remain the same or even decrease a bit until it is confirmed that the peak has passed. Then *β*_n_ will increase because the interventions for social distancing and wearing face masks will be gradually lifted. Furthermore, the new Omicron subvariants with greater infectivity may spread any day after $\bar{y}$_n_ peak to increase $\bar{y}$_n_ again. These unknown or undetermined factors make it almost impossible to make long-term prediction of the exact trajectory of $\bar{y}$_n_. However, since *β*_n_ most likely reaches its minimum value around the $\bar{y}$_n_ peak, if we use this minimum value of *β*_n_ to predict changes in $\bar{y}$_n_ in the near future, the simulated *y*_n_ curve will be likely lower than the reported $\bar{y}$_n_ curve. This enables us to predict the lower bound of $\bar{y}$_n_ curve in the near future after the $\bar{y}$_n_ peak. The lower bound of $\bar{y}$_n_ curves (dashed line) in Fig 3 was obtained by assuming that *β*_n_ = 0.16/day after January 22, 2022. In addition to calculating the lower bound of *y*_n_ curve, we can also calculate an upper bound of the $\bar{y}$_n_ curve by assuming that *β*_n_ rapidly increases to 1 or a greater number in a short period of time (solid line in Fig 3). This period is chosen to be significantly shorter than the actual period needed to increase *β*_n_ in the real world. In the calculations, we assumed that no new Omicron subvariants appear in this time period to affect $\bar{y}$_n_ largely. As shown in Fig 3, the reported daily COVID-19 cases ($\bar{y}$_n_) were within the predicted lower and upper bounds for 3 months, until April 22, 2023.

### Simulating Omicron sub-variants induced increases in ${\bar{y}}_{n}$

**Fig 4:**
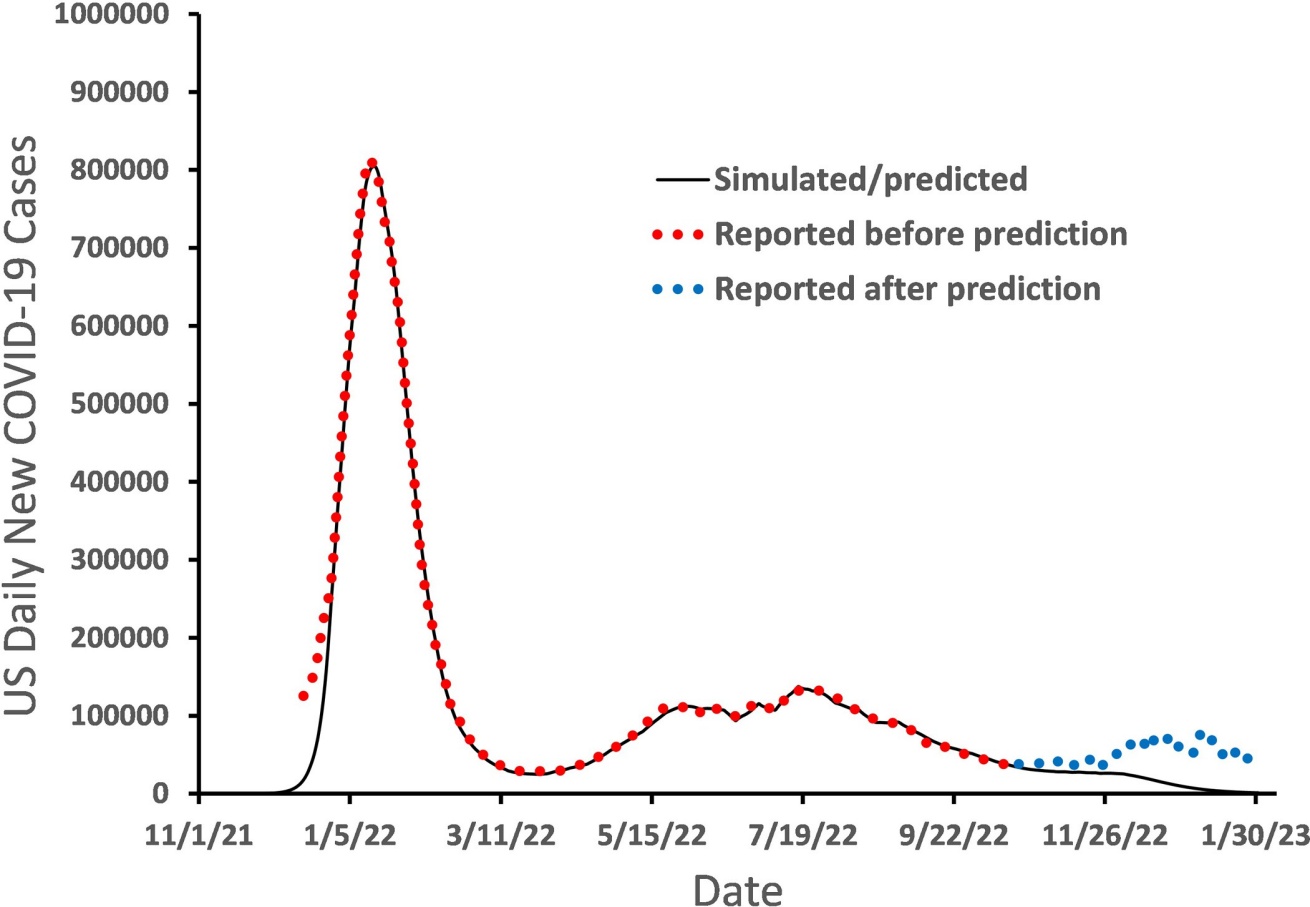
Simulated and reported number of Omicron-caused daily new COVID-19 cases in the United States without considering Omicron reinfection in the model.

After the Omicron-caused large peak of COVID-19 transmission in the US, the reported number of daily new COVID-19 cases, $\bar{y}$_n_, gradually decreased until early April 2022. Then, $\bar{y}$_n_ started to increase again because of the increased *β*_n_ and transmission of multiple new Omicron subvariants with higher contagiousness in comparison to the original Omicron strain B.1.1.529 (other names: B.1.1.529.1 or BA.1) [39, 40]. The Omicron and its major sub-variants that had significant contributions to COVID-19 transmission in the United States include (BA.1 & BA.1.1), BA.2, BA.2.12.1, BA.2.75, BA.4, BA.4.6, BA.5, and (BQ.1 & BQ.1.1) [41, 42]. Among these sub-variants, BA.1 and BA.1.1 were dominated in the big peak of $\bar{y}$_n_ as of mid-February 2022 [43], and then other sub-variants followed separately. Considering that the later Omicron sub-variants had larger infectivity, we assume that each new sub-variant mentioned above can affect COVID-19 transmission by enlarging the number of susceptible people *N*. To simulate the Omicron-caused changes in *y*_n_ after mid-February 2022, we allowed *N* to increase on some selected dates (from *N* = 250,000,000 to *N* = 332,400,000) between the end of 2021 and early October 2022, while *β*_n_ gradually increases to 1 as of mid-September 2022. In this way, the simulated *y*_n_ can fit the reported $\bar{y}$_n_ very well (Fig 4) as of Oct 23, 2022. It needs to be noted that, when *N* increases to 332400000, almost all of the population in the US has become susceptible to the highly infectious Omicron sub-variants. To predict *y*_n_ after October 23, 2022, we let *β*_n_ continuously increase to 3.5 before the end of November 2022, and remain at 3.5 after November 2022. The predicted *y*_n_ (solid line) forms a plateau from late October 2022 to the end of November 2022, and then *y*_n_ significantly decreases after early December, 2022, and *y*_*n*_ drops to nearly 1000 cases/day by the end of January 2023. This predicted result was uploaded to Twitter in late October 2022 [44]. The reported daily COVID-19 cases met the predicted results well until early December 2022 [45]. Our simulation and prediction showed that after August 2022, especially after the *y*_n_ plateau in early December 2022, increasing *β*_n_ or emergence of more contagious Omicron variants would not push *y*_*n*_ up. This implies that the herd immunity to omicron has been reached in the United Sates base on the *l-i SEIR* model. In the above *l-i SEIR* model, it was assumed that any individual infected by an Omicron sub-variant would not be reinfected by any other Omicron subvariants and any new COVID-19 variants. However, in reality, Omicron-infected individuals still have a chance to be reinfected by an Omicron subvariant, even though the reinfection chance is very low. Therefore, the infected people are not able to form a perfect herd immunity. As we have seen, the reported daily new COVID-19 cases after late October 2022 (blue dots) formed a plateau between late October 2022 and late November 2022, which agreed with the predicted curve very well. However, the reported daily new COVID-19 cases slightly increased in the period between December 2022 and January 2023 because of the social gatherings in the holiday seasons (Christmas and New Year); and a more contagious Omicron variant XBB.1.5 also appeared in this period [46]. This deviation from the predicted curve based on *l-i SEIR* model implies that a small ratio of Omicron-infected people can be re-infected by Omicron sub-variants and that the Omicron-to-Omicron reinfection needs to be considered in the modelling.

### Simulating and predicting the trajectory of $\bar{y}$_n_ in the presence of reinfection of Omicron infections

**Fig 5:**
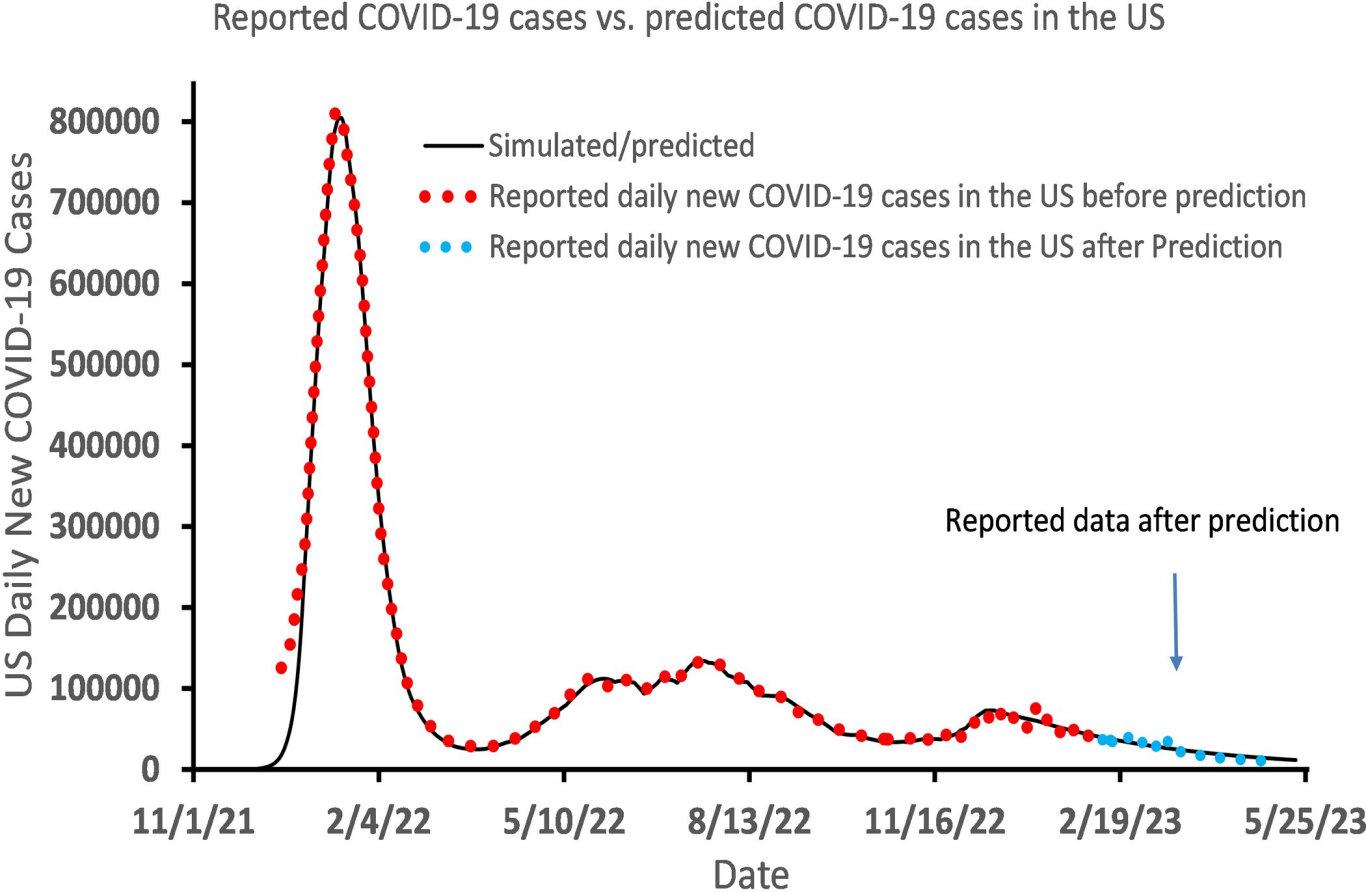
Simulated and predicted and reported number of Omicron-caused daily new COVID-19 cases in the United States after considering Omicron reinfection in the model.

In the above *l-i SEIR* model, the number of susceptible people *S*_*n*_ varies between 0 and *N*, or *0*≤*S*_*n*_≤*N*. If most of susceptible people have been infected, then S_*n*_ is far smaller than *N* and the ratio *S*_*n*_/*N* is near zero. Therefore, the number of daily new exposed people, (*S*_*n*_*-S*_*n-*1_) in Eqn (1a), is also near 0. However, if the rate of reinfection of Omicron infected people is non-negligible, (*S*_*n*_*-S*_*n-*1_) must not be near zero even if all susceptible people have been infected. Thus, we suggest that in the presence of non-negligible rate of reinfection, the ratio *S*_*n-*1_*/N* in Eqn (1a) should be replaced by [*S*_*n-*1_(≥0)*/N* + *b*_*n*_(*N*- *S*_*n-*1_(≥0))/*N*] as shown in Eqn (1a)’. Based on Eqns (1a)’ and (1b)–(1d), we simulated and predicted daily new COVID-19 cases on February 10, 2023 [47] assuming that *b*_*n*_ = 0.03, and compared them with later reported data until May 5, 2023 (Fig 5) [48] when data of daily COVID-19 cases in the US were not updated anymore on websites. The red dots in Fig 5 represent the number of daily COVID-19 cases (${\bar{y}}_{n}$) reported before the predicted trajectory of *y*_*n*_ (solid black line) was generated. The blue dots in Fig 5 represent ${\bar{y}}_{n}$ reported after the predicted trajectory of *y*_*n*_ (solid black line) was generated. The result in Fig 5 shows that the reported ${\bar{y}}_{n}$ matches very well with the predicted trajectory of *y*_*n*_, indicating that we successfully achieved a nearly 3-month long prediction of trajectory of the reported daily COVID-19 cases (${\bar{y}}_{n}$) in the USA.

### Summary

Based on the *l-i SEIR* model, the authors described difficulties and discussed possible solutions in forecasting the peak date and the peak height of daily new COVID-19 cases (${\bar{y}}_{n}$) caused by Omicron, the trajectory of ${\bar{y}}_{n}$ after the ${\bar{y}}_{n}$ peak, and the trajectory of ${\bar{y}}_{n}$ after the herd immunity was reached in the presence or absence of Omicron-to-Omicron reinfection. Our simulations show that by using the *β*_n_ determined from the latest reported ${\bar{y}}_{n}$ data, one may predict the date of ${\bar{y}}_{n}$ peak within a limited prediction error, and also predict an upper limit for the height of the ${\bar{y}}_{n}$ peak. It is possible to accurately predict the trajectory of *y*_*n*_ after the ${\bar{y}}_{n}$ peak for a few weeks (up to 4 weeks from 1/22/2022-2/19/2022 as shown in Fig 3) with a constant *β*_n_. However, by calculating a lower limit and an upper limit of the *y*_*n*_ curve, one may successfully predict the trace of ${\bar{y}}_{n}$ within the range between the lower limit and upper limit of the *y*_*n*_ curve for more than 3 months (from 1/22/2022 to 4/28/2022 in Fig 3). The *l-i SEIR* model without considering Omicron-to-Omicron reinfection could not explain the remaining non-negligible number of daily new COVID-19 cases after the herd immunity was reached (*S*_*n*_/*N*≈0), suggesting that the Omicron-to-Omicron reinfection should be taken into account in the model. The simulated *y*_*n*_ curve based on the *l-i SEIR* model considering Omicron-to-Omicron reinfection can fit very well with the numbers of reported COVID-19 cases after the herd immunity has been reached, and the predicted *y*_*n*_ curve is in good agreement with the number of daily new COVID-19 cases reported as of May 10, 2023, twelve weeks after the prediction of *y*_*n*_ curve was made on February 10, 2023.
